# Notifications and alerts in patient dose values for computed tomography and fluoroscopy-guided interventional procedures

**DOI:** 10.1007/s00330-022-08675-w

**Published:** 2022-03-16

**Authors:** Eliseo Vano, Reinhard Loose, Guy Frija, Graciano Paulo, Efstathios Efstathopoulos, Claudio Granata, Riccardo Corridori, Alberto Torresin, Jonas S. Andersson, Virginia Tsapaki, Josefin Ammon, Christoph Hoeschen, On behalf of the European Society of Radiology

**Affiliations:** 1 Radiology Department, Complutense University, 28040 Madrid, Spain; 2 Institute of Medical Physics, Hospital Nuremberg, Prof.-Ernst-Nathan-Str. 1, 90419 Nuremberg, Germany; 3 Université de Paris, 12 Rue de l’École de Médecine, 75006 Paris, France; 4 Medical Imaging and Radiotherapy Department, Instituto Politécnico de Coimbra, ESTESC-Coimbra Health School, Rua 5 de Outubro, S. Martinho do Bispo, 3046-854 Coimbra, Portugal; 5 Department of Radiology, Medical Physics Unit, National and Kapodistrian University of Athens, Attikon University Hospital, 12462 Athens, Greece; 6 Institute for Maternal and Child Health, IRCCS “Burlo Garofolo”, Trieste, Italy; 7 COCIR, 80 Bd. A. Reyers, 1030 Brussels, Belgium; 8 Medical Physics Department, Ospedale Niguarda, Milan, Italy; 9 Department of Radiation Sciences, Umea University, Umeå, Sweden; 10 Medical Physics, Konstantopoulio General Hospital, Nea Ionia, Greece; 11 Institute of Medical Physics, Nuremberg General Hospital, Paracelsus Medical University, Nuremberg, Germany; 12 Institut Für Medizintechnik, Otto-Von-Guericke Universität, Magdeburg, Germany

**Keywords:** Notification, Alert, Tomography, X-ray computed, Fluoroscopy-guided interventional procedures, Unintended exposures

## Abstract

**Abstract:**

The terms “notifications” and “alerts” for medical exposures are used by several national and international organisations. Recommendations for CT scanners have been published by the American Association of Physicists in Medicine. Some interventional radiology societies as well as national authorities have also published dose notifications for fluoroscopy-guided interventional procedures. Notifications and alerts may also be useful for optimisation and to avoid unintended and accidental exposures. The main interest in using these values for high-dose procedures (CT and interventional) is to optimise imaging procedures, reducing the probability of stochastic effects and avoiding tissue reactions. Alerts in X-ray systems may be considered before procedures (as in CT), during procedures (in some interventional radiology systems), and after procedures, when the patient radiation dose results are known and processed. This review summarises the different uses of notifications and alerts to help in optimisation for CT and for fluoroscopy-guided interventional procedures as well as in the analysis of unintended and accidental medical exposures. The paper also includes cautions in setting the alert values and discusses the benefits of using patient dose management systems for the alerts, their registry and follow-up, and the differences between notifications, alerts, and trigger levels for individual procedures and the terms used for the collective approach, such as diagnostic reference levels.

**Key Points:**

• *Notifications and alerts on patient dose values for computed tomography (CT) and fluoroscopy-guided interventional procedures (FGIP) allow to improve radiation safety and contribute to the avoidance of radiation injuries and unintended and accidental exposures*.

• *Alerts may be established before the imaging procedures (as in CT) or during and after the procedures as for FGIP*.

• *Dose management systems should include notifications and alerts and their registry for the hospital quality programmes*.

## Introduction

The terms “notifications” and “alerts” for some medical exposures are being used by standardisation organisations, by the Food and Drug Administration (FDA), and by several interventional radiology societies to provide information of certain radiation quantities in relation to patient doses [1–5].

A United States (US) technical standard (XR 25) published by the National Electrical Manufacturers Association (NEMA) in 2010 [1] has been used by the American Association of Physicists in Medicine (AAPM) to produce a set of “Recommendations Regarding Notification and Alert Values for CT Scanners” [3]. Also, the International Electrotechnical Commission (IEC) has included these terms in some of its documents [4] and it is expected that the definitions will be improved in the future. Dose notification and alert values for CT are based on computed tomography dose index (CTDIvol), which are related to a constant phantom size, independent of the real patient size.

The North American Society of Interventional Radiology (SIR) and the Cardiovascular and Interventional Radiology Society of Europe (CIRSE) published a set of dose notifications for fluoroscopy-guided interventional procedures (FGIP) [5].

Notifications and alerts may also be useful for optimisation to avoid “unintended and accidental exposures”. This is required by the European Basic Safety Standards directive [6]. A specific section on alarms for these exposures is also included in this review.

Computed tomography (CT) scanners in compliance with the NEMA standard can be configured to inform users when scan settings would yield values of CTDIvol and dose length product (DLP) that would exceed pre-assigned values. Compliant scanners allow users to confirm or correct settings that might otherwise lead to unnecessarily high exposures, before proceeding with scanning. Manufacturers may include pre-assigned values in their default protocols, but all values are user-configurable [3].

The primary purpose of notification and alert values for CT, as intended by the FDA in their request to manufacturers, is to prevent relevant errors at the time of the patient scan. It is also important to develop criteria regarding the level of detail that is required to be included in the event registry, as well as defining which member(s) of a practice are authorised to approve to proceed with a scan where CTDIvol and DLP would exceed the alert level [7].

The main interest in using these values for high-dose procedures (CT and FGIP) is the avoidance of radiation skin injuries, but alerts in X-ray systems may be considered before procedures (as in CT), during procedures (as in some interventional radiology systems), and after procedures, when the patient dose descriptor results are known and processed. High patient dose values may also involve high occupational doses and this should be considered in optimisation.

There are few reported experiences on the practical use of these notifications and alerts in CT and their impact on the workflow of radiology departments and on the optimisation of radiation protection of patients. Howard et al [7] reported a 1.2% level for triggering a notification for CT examinations (large patient size and bolus tracking). However, authors note that the low percentage of alerts found was due to three factors: the review process of the protocols, an optimised practice in large patients, and the use of only new scanners.

There are more publications and international recommendations that deal with radiation skin injuries in FGIP [8, 9].

Alerts and trigger levels are useful tools to optimise imaging procedures, reducing the probability of stochastic effects and avoiding tissue reactions (deterministic effects).

In diagnostic and interventional radiology, the two terms (notifications and alerts) and sometimes other terms such as “threshold value” or “significant dose events” are used to inform about a high (or potentially high) skin dose for the patient or for unintended exposures. Sometimes, other patient organs with a risk of radiation injury, such as the lenses of the eyes and the cerebrovascular or cardiovascular systems, may be also considered [10–13].

This review summarises the different uses of notifications and alerts to help in optimisation for CT and for fluoroscopy-guided interventional procedures as well as in the analysis of unintended and accidental medical exposures. The paper includes cautions in setting the alert values and discusses the benefits of using patient dose management systems for the alerts, their registry and follow-up, and the differences between alerts for individual procedures and diagnostic reference levels (DRLs).

## CT systems

The most advanced prospective use of notifications and alerts, used before carrying out a procedure, has already been implemented by the radiology industry and is being applied in CT systems. It is used to alert the operator when selecting one specific protocol with CTDIvol or DLP higher than the standard protocol. In these cases, the notifications and alerts are presented prior to the examinations and the operator has the possibility to modify or correct the selected protocol if there is a mistake.

The NEMA standard XR-25 and the AAPM [3] employ the following definitions:

- **Notification value**: A value of CTDIvol (in units of mGy) or DLP (in units of mGy.cm) used to trigger a notification when the value would likely be exceeded by the prescribed scans. When the system projects that a notification value will be exceeded for any of the prescribed scans, the user is notified via a pop-up window prior to scanning and is required either to verify that the settings are correct or to change them.

The AAPM Working Group on Standardization of CT Nomenclature and Protocols, which includes members from the FDA, American College of Radiology (ACR), and manufacturers, established in 2011 a particular set of notification values (see Table 1). These values may be refined in ulterior publications as the Adult Brain Perfusion CT Protocols version 2.0 3/1/2016 [14].

**Table 1:** Notification values recommended by the AAPM Working Group on Standardization of CT Nomenclature and Protocols [3]

CT scan region (of each individual scan in an examination)	CTDIvol notification value (mGy)
Adult head	80
Adult torso	50
Pediatric head	
< 2 years old	50
2–5 years old	60
Pediatric Torso	
< 10 years old (16-cm phantom)	25
< 10 years old (32-cm phantom)	10
Brain perfusion	
(examination that repeatedly scans the same anatomic level to measure the flow of contrast media through the anatomy)	600
Cardiac	
Retrospectively gated (spiral)	150
Prospectively gated (sequential)	50

- **Alert Value**: A value of CTDIvol or DLP used to trigger an alert when the system projects that the prescribed scans within an ongoing examination would result in a cumulative dose index value that exceeded the user-configured alert value. The cumulative dose index value is compared to the alert value at each anatomic position throughout an examination. While any individual scan might not trigger a notification or alert, if the cumulative dose index value at any anatomic position were expected to exceed the alert value when the next scan was performed, an alert would be triggered prior to scanning.

An alert value is associated with a complete examination protocol, not with individual scans. An alert warrants more stringent review before proceeding and requires a higher level of action by the user.

The FDA has suggested an alert value for CTDIvol of 1000 mGy, which would deliver approximately half the dose associated with the onset of skin injury [2].

It might be noted that the CT section of the Digital Imaging and Communications in Medicine (DICOM) Radiation Dose Structured Report (RDSR) provides fields for dose notifications and dose alerts [4].

## Interventional fluoroscopy systems

For FGIP, the approach is more complex. This is because the patient skin dose depends not only on the patient weight and the automatic exposure control system, but also on the selected image acquisition mode (fluoroscopy mode low, medium, or high, cine mode, digital subtraction angiography (DSA), cone beam CT (CBCT), etc.), C-arm angulations, collimation, and other geometrical parameters.

In modern fluoroscopy X-ray systems, the information on patient dose and patient dose rate is displayed during the procedures in terms of air kerma area product—*P*_KA_—and cumulative air kerma—*K*_ar_—at the patient entrance reference point. Interventionists are thus able to modify the operational/technical protocol of the procedure to optimise the procedure (e.g. avoiding some cine or DSA series, storing fluoroscopy runs, modifying the C-arm angulations, and using field collimation to avoid overexposure of a patient skin region).

Sometimes, additional calculations need to be made by a medical physics expert if skin dose mapping software is not available. In most cases, the final patient dose values are considered to be achieved at the end of the procedures. It is necessary to evaluate the impact of the procedure’s complexity and to consider if optimisation actions may be used in other similar situations.

Particularly relevant are alerts for skin dose values exceeding the thresholds, to properly manage follow-up processes. Validated methods for evaluation of this metric are necessary [15, 16].

The DICOM RDSR, already available in most of the modern X-ray interventional systems, allows the registration and processing of many geometrical, operational, and dosimetric parameters to set notifications and alert levels for some abnormal situations (e.g. have the image detector far from the patient or have the X-ray tube very close to the patient in horizontal projections).

Some interventional X-ray systems may offer notifications and alerts in real time when dosimetric values are higher than a certain value. The SIR-CIRSE recommendations [5] suggest some values for notification levels (see Table 2). In any case, the result on patient skin dose (and “peak skin dose”, if available) is usually obtained at the end of the interventional procedure. For X-ray units with information on the *K*_a,r_, initial notification is given at 3000 mGy and then every 1000 mGy thereafter. These values may correspond to an initial peak skin dose of about 1800 mGy and an increment of about 500 mGy. For units with *K*_a,r_ capability, the notification level is based on a procedure-dependent nominal X-ray field size at the patient’s skin. Using a 100 cm^2^ field, the initial report would be at 300 Gy.cm^2^ and subsequently at increments of 100 Gy.cm^2^ [5].

**Table 2:** Summary of radiation monitoring dose notification thresholds [5]

Parameter	First notification	Subsequent notifications
Peak skin dose (PSD)	2000 mGy	500 mGy
Reference point air kerma (*K*_a,r_)	3000 mGy	1000 mGy
Kerma area product (*P*_KA_)	300 Gy.cm^2^ (*)	100 Gy.cm^2^ (*)
Fluoroscopy time (FT)	30 min	15 min

(*) Assuming a 100 cm^2^ field at the patient’s skin. The value should be adjusted to the actual procedural field size

According to Miller et al [17], some dosimetric aspects should be considered as part of the pre-procedure patient evaluation (e.g. if in some previous procedures a part of the skin could have received high doses, this should be known before starting a new procedure and re-irradiating that skin region should be avoided). Dose optimisation is possible through appropriate use of the basic features of interventional fluoroscopic equipment and intelligent use of dose-reducing technology.

Information on occupational doses (and dose rates) may also be available inside the interventional room if new types of electronic personal dosimeters are available. This allows setting “notifications” or “alerts” for occupational doses to implement active optimisation in real time (e.g. if the ceiling suspended screen is not used or is in the wrong position) [18].

## Alerts for unintended and accidental medical exposures

The European Directive on BSS [6] requires notification and reporting of unintended and accidental medical exposures. These events are defined as “medical exposure that is significantly different from the medical exposure intended for a given purpose”.

The European Society of Radiology (ESR) published a white paper in 2019 [19] with the results of a survey among ESR member countries including all EU MS highlighting a very heterogeneous and unsatisfactory situation in transposition of the EU-BSS. ESR recommends notifications and reporting criteria for significant events based on physical quantities and units and not on effective dose [20].

The Working Group “Dosimetry in imaging for clinical practice” of EuroSafe Imaging recommended the use of trigger levels for individual patients, to avoid skin injuries [20].

A “trigger level” is an appropriately selected reference value, usually of the cumulative air kerma (*K*_a,r_), indicating an increased risk of tissue reactions on the skin [13].

In this paper, the ESR refers to the “individual approach” (unnecessary overexposures of individual patients with the risk of deterministic effects, high effective doses, or high organ doses) and “collective approach” (group of patients with the risk of stochastic effects). The collective approach involves exposure of a larger number of patients with low doses well below the threshold of deterministic effects.

Loose et al published a detailed analysis of the radiation protection framework since 2019, referring to the implementation of the European Directive in Germany and a comparison with seven European countries, including the aspects of unintended exposure of patients [21]. The paper contains a table comparing the reporting criteria in Germany, Ireland, UK, Spain, Belgium, Switzerland, and Austria.

The criteria for reporting significant events in radiology in Germany are different for individual patients and for a group of patients.

- For CT and FGIP, the collective approach for groups of patients uses 3 times the amount of the DRL as a trigger level. A reporting threshold refers to the 20 previously performed identical procedures using the identical device, if the average of these 20 procedures exceeds the DRL by 100%.
- For individual patients, the CT threshold values for CTDIvol are 120 mGy and 80 mGy for brain and body respectively and for fluoroscopy, *P*_KA_ threshold values of 200 and 500 Gy.cm^2^ for diagnostic and interventional procedures, respectively.

Jaschke et al have summarised some aspects of the “unintended and accidental exposures” referring to the significant dose events and trigger levels in FGIP [13]. Notification or alert levels may be set below the trigger level in order to notify the interventionist that he/she is approaching the trigger level so that further optimisation of the procedure can be considered at an earlier stage.

If an alert or trigger level is exceeded while performing an FGIP, the interventionist can decide whether to modify the technical parameters of the imaging protocol or the interventional procedure to avoid excessive radiation exposure to the patient, or to continue with the procedure if the benefit for the patient outweighs the risk. If patient dose is at, or above a trigger level, then the interventionist shall place a note of the exposure in the medical record after completing the procedure, and the patient should be followed up with.

Martin et al also refer to the unintended and accidental medical radiation exposures in radiology [22] in relation to the overexposures in skin to produce tissue reactions, in FGIP and CT, most notably from perfusion studies.

Unfortunately, not all the “unintended and accidental exposures” will be easily detected with a notification or an alert in patient dose (e.g. imaging of a wrong body part or imaging the wrong patient). Crowley et al have described the advantages of a radiographer feedback to help in the management of dose alerts [23].

Table 3 presents a summary of the used terms: notification, alert, trigger level, and DRL.

**Table 3:** Definitions summary

Notification value (usually used for CT) [3]	A value of CTDI vol or DLP to trigger a notification when the value would likely be exceeded by the prescribed scans. The user is required to verify that the settings are correct or to change them.
Alert values (usually used for CT) [3]	A value of CTDIvol or DLP used to trigger an alert when the system projects that the prescribed scans within an ongoing examination would result in a cumulative dose index value that exceeded the user-configured alert value. An alert value is associated with a complete examination protocol, not with individual scans.
Trigger level (for interventional fluoroscopy systems and for unintended exposures) [13]	A “trigger level” is an appropriately selected reference value, usually of the cumulative air kerma (*K*_a,r_), indicating an increased risk of tissue reactions on the skin.
Diagnostic reference level (not for individual patients) [24]	Used in medical imaging to indicate whether, in routine conditions, the amount of radiation used for a specified procedure is unusually high or low for that procedure.

## Discussion

If the notifications and/or alerts are very frequent (e.g. if setting at very low dose values), operators could not consider them. A few percent (0.1 to 1.0%) can be considered reasonable for CT systems [7].

Dose management systems (DMS) should be able to extract statistical information related to procedures with patient doses higher than certain values. This information should be used to set the notification and alert levels and to implement corrective actions [25].

If the DMS is able to estimate cumulative effective doses (CED) in recurrent examinations in the same patient, some alert levels could also be derived from it to help in refining justification and optimisation of future procedures in these patients. One limitation for these estimations of cumulative doses is that they are only reliable with a central dose repository system if all procedures are not performed in a single institution. The uncertainties in the estimations of effective dose should always be considered as well as the limits of the concept of effective dose for medical applications.

It is important to differentiate the alerts for a single procedure (when patient dose values may be very high) and the alerts for the tendency, e.g. by comparing the median value of a group of procedures for the same clinical indication, with DRLs values.

ICRP has recommended that DRLs should not be used as alert levels. However, in practice, sometimes a large deviation from the local DRLs may be used as an easy way to alert of an overexposure.

A DMS could analyse the median values of groups of procedures to detect an increase in median dose values, compare them with DRLs, and alert the operators if median dose values may be higher than the DRLs. In addition, some criteria for additional single alerts may be considered (e.g. if an individual patient dose value, for standard sized patients, could be higher than 2–3 times the DRLs) [25].

## Conclusions and practical recommendations

- Notification and alert dose values may be useful for optimisation and to avoid, in some cases, unintended or accidental exposures. High patient dose values may also involve high occupational doses and this should be considered in optimisation.
- A large percentage of notifications and alerts should be avoided to maintain the attention of the operators on the workflow.
- Notification and alert values may be used prospectively (as is already possible in some CT systems) and retrospectively, considering the dosimetric parameters at the end of FGIPs. New interventional X-ray systems also offer continuous dosimetric information during the procedures, allowing active optimisation.
- Peak skin dose and skin dose maps, properly calculated, are one of the best tools to optimise radiation protection in complex fluoroscopy-guided interventions to avoid radiation injuries.
- The use of the statistical analysis from dose management systems allows setting notification and alert levels for different procedures, continuous comparison with local DRLs, and registration of the occurring alerts.
- In any case, the priority should always be the clinical setting and outcome and an appropriate level of image quality and diagnostic information for the procedures.

## Abbreviations

AAPM: American Association of Physicists in Medicine
ACR: American College of Radiology
BSS: Basic Safety Standards
CBCT: Cone beam computed tomography
CED: Cumulative effective dose
CIRSE: Cardiovascular and Interventional Radiology Society of Europe
CT: Computed tomography
CTDIvol: Computed tomography dose index volumetric
DICOM: Digital Imaging and Communications in Medicine
DLP: Dose length product
DMS: Dose management systems
DRLs: Diagnostic reference levels
DSA: Digital subtraction angiography
EU: European Union
FDA: Food and Drug Administration
FGIP: Fluoroscopy-guided interventional procedures
ICRP: International Commission on Radiological Protection
IEC: International Electrotechnical Commission
*K*_ar_: Cumulative air kerma at the patient entrance reference point
MS: Member states (of the European Union)
NEMA: National Electrical Manufacturers Association
*P*_KA_: Air kerma area product
RDSR: Radiation Dose Structured Report
RIS: Radiology Information System
SIR: Society of Interventional Radiology
US: United States of America

## Declarations

### Guarantor

The scientific guarantor is Eliseo Vano.

### Statistics and biometry

No complex statistical methods were necessary for this paper.

### Methodology

•Review article
